# High Production of 3-Hydroxypropionic Acid in *Klebsiella pneumoniae* by Systematic Optimization of Glycerol Metabolism

**DOI:** 10.1038/srep26932

**Published:** 2016-05-27

**Authors:** Ying Li, Xi Wang, Xizhen Ge, Pingfang Tian

**Affiliations:** 1 Beijing Key Laboratory of Bioprocess, College of Life Science and Technology, Beijing University of Chemical Technology, Beijing 100029, People’s Republic of China; 2 College of Biochemical Engineering, Beijing Union University, Beijing 100023, People’s Republic of China

## Abstract

3-Hydroxypropionic acid (3-HP) is an important platform chemical proposed by the United States Department of Energy. 3-HP can be converted to a series of bulk chemicals. Biological production of 3-HP has made great progress in recent years. However, low yield of 3-HP restricts its commercialization. In this study, systematic optimization was conducted towards high-yield production of 3-HP in *Klebsiella pneumoniae*. We first investigated appropriate promoters for the key enzyme (aldehyde dehydrogenase, ALDH) in 3-HP biosynthesis, and found that IPTG-inducible *tac* promoter enabled overexpression of an endogenous ALDH (PuuC) in *K. pneumoniae*. We optimized the metabolic flux and found that blocking the synthesis of lactic acid and acetic acid significantly increased the production of 3-HP. Additionally, fermentation conditions were optimized and scaled-up cultivation were investigated. The highest 3-HP titer was observed at 83.8 g/L with a high conversion ratio of 54% on substrate glycerol. Furthermore, a flux distribution model of glycerol metabolism in *K. pneumoniae* was proposed based on *in silico* analysis. To our knowledge, this is the highest 3-HP production in *K. pneumoniae*. This work has significantly advanced biological production of 3-HP from renewable carbon sources.

**T**he depletion of oil reserves and the deterioration of the environment have necessitated biorefinery as an alternative to chemical synthesis. 3-Hydroxypropionic acid (3-HP) is one of the top 12 value-added platform compounds among renewable biomass products proposed by the United States Department of Energy^1^. Currently, 3-HP is gaining increased interest because of its versatile applications. For instance, 3-HP can be easily converted to a range of bulk chemicals, such as acrylic acid, 1,3-propanediol (1,3-PD), 3-hydroxypropionaldehyde (3-HPA), and malonic acid. In addition, 3-HP can be polymerized to form biodegradable materials^2^,^3^. To date, diverse microbes that are able to naturally produce 3-HP have been identified^4^. *Klebsiella pneumoniae* is of great attractiveness owing to the striking biological attributes, such as remarkable capacity to metabolize glycerol, active cell proliferation, and particularly the native capability to synthesize vitamin B_12_. Importantly, vitamin B_12_ serves as the cofactor of glycerol dehydratase^5^. It is clear that *K. pneumoniae* as a host is superior to *E. coli* which needs the addition of expensive vitamin B_12_ to fermentation medium during 3-HP biosynthesis^6^,^7^.

Glycerol metabolism in *K. pneumoniae* is mediated by the *dha* regulon which governs glycerol oxidation and reduction pathways in parallel (Fig. 1)^8^. In the glycerol oxidation pathway, glycerol is converted to dihydroxyacetone phosphate (DHAP) which subsequently enters glycolytic pathway. In the glycerol reduction pathway, glycerol is converted to 3-HPA by glycerol dehydratase (EC: 4.2.1.30, GDHt, encoded by *dhaB* gene cluster, GenBank U30903), and then 3-HPA can be converted to 1,3-propanediol by 1,3-propanediol oxidoreductase (DhaT, EC:1.1.1.202) and 3-HP by aldehyde dehydrogenase (ALDH, NAD^+^-dependent, EC:1.2.1.-), respectively (Fig. 1)^9^,^10^.

Among the enzymes participating in glycerol dissimilation in *K. pneumoniae*, GDHt is the first enzyme catalyzing reaction in the reduction pathway^8^,^11^. The molecular modification, deletion, or overexpression of GDHt has not been well documented presumably because it is a well-evolved, high-activity, and multisubunit enzyme tailored for the conversion of glycerol to 3-HPA. 3-HPA is toxic to cells, and is naturally converted towards 1,3-PD by a high-activity enzyme DhaT. In sharp contrast to DhaT, the native ALDHs exhibit low activities in *K. pneumoniae*. Therefore, only trace amount of 3-HP can be detected in wild-type *K. pneumoniae*. Clearly, ALDH is the velocity-limiting enzyme for the production of 3-HP^7^,^12^,^13^,^14^. Thus, to overproduce 3-HP, a high-activity ALDH is highly desirable. Among numerous ALDHs reported so far, PuuC (GenBank: YP_001334683), a native ALDH of *K. pneumoniae*, displayed pronounced activity towards 3-HPA^15^.

In addition to ALDH activity, promoter strength is also critical for the 3-HP production. Previously, heterologous expression of ALDH in *K. pneumoniae* was accomplished mainly by harnessing constitutive promoter, e.g. *lac* or *pk*, which *pk* is the native promoter of *dhaB1* genes, the first subunit of *dhaB* gene cluster in *K. pneumoniae* DSM 2026^8^. Due to the innate low activities of constitutive promoters, this method was not successful for the overproduction of 3-HP. Considering *tac* promoter is functional in *E. coli*, *Pseudomonas putida* and *Ralstonia* sp upon the induction of isopropylthio-β-d-galactoside (IPTG)^16^,^17^,^18^, we hypothesized that *tac* promoter might also work well with the transcription machinery in *K. pneumoniae*.

Apart from promoter and enzyme activities, cell growth and cofactor availability are also critical to 3-HP biosynthesis. For example, 3-HP biosynthesis was shown closely coupled with cell proliferation especially at the exponential phase^19^. In addition, 3-HP production is largely affected by NAD^+^ and NADH, which serve as the cofactors of ALDH and DhaT, respectively. In fact, there could be a competition between ALDH and DhaT for the substrate 3-HPA on the availability of their related cofactors. On the other hand, NAD^+^ and NADH also serve as the cofactors of lactate dehydrogenase (EC:1.1.2.3) and phosphate acetylasetransferase, which respectively catalyze the formation of lactic acid and acetic acid, the major byproducts in the production of 3-HP. Thus, it is clear that the cofactors can significantly affect 3-HP production by interfering glycerol oxidation and reduction reactions in *K. pneumoniae*.

In view of above information, we reasoned that 3-HP production relies upon multiple factors, including enzyme activity, promoter strength, cell growth, ATP provision, and cofactor availability. Following this assumption, systematic optimization was conducted to improve the 3-HP production in *K. pneumoniae*, including screening of appropriate promoter, modification of metabolic pathway, amelioration of medium composition, and optimization of fermentation conditions such as inducer concentration, pH value and dissolved oxygen (DO). In particular, we compared the constitutive *lac* promoter and inducible *tac* promoter for their effects on ALDH activity and 3-HP production. Finally, under optimized conditions, the most competitive recombinant *K. pneumoniae* strain was cultivated in a 5 L bioreactor to evaluate the 3-HP production.

## Results

### Screening of appropriate promoter for production of 3-HP

**Figure 2:**
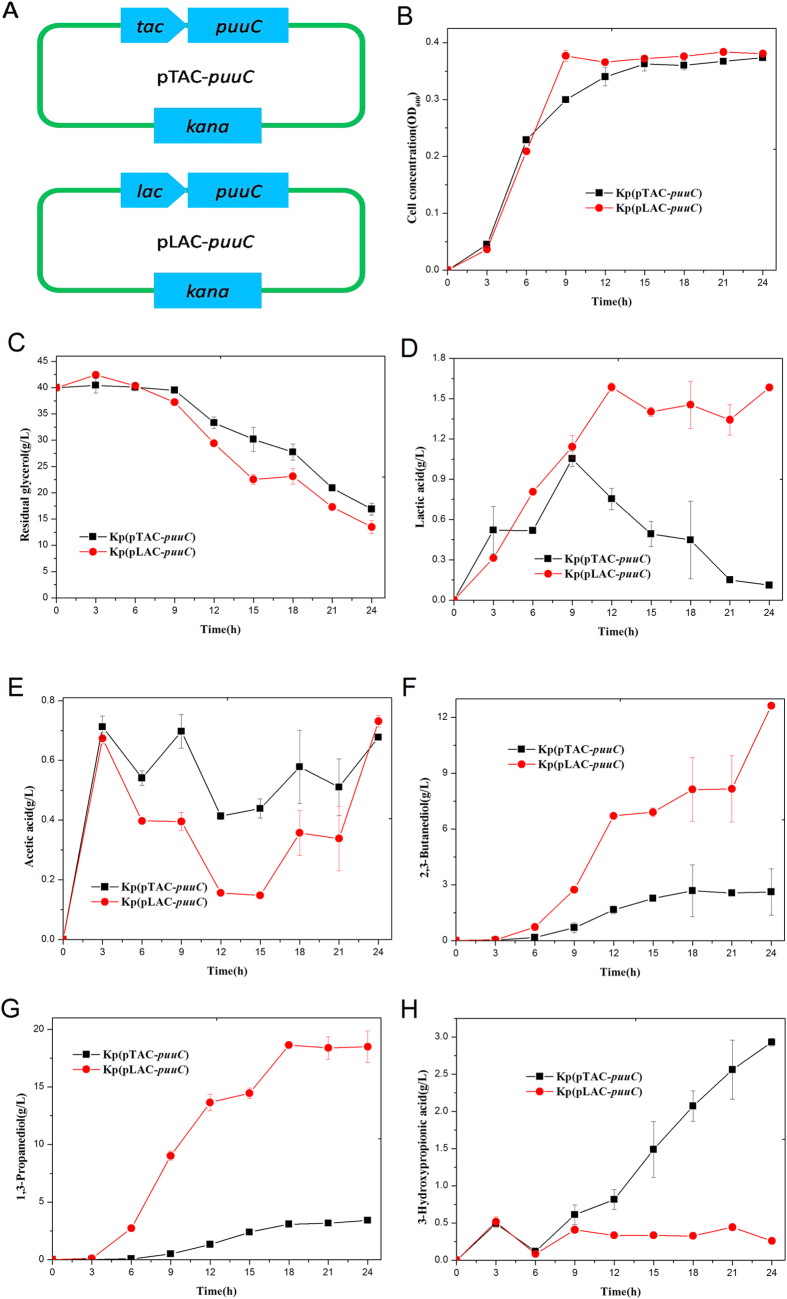
Comparison of *tac* and *lac* promoters for their efficiency driving PuuC expression and accordingly the 3-hydroxypropionic acid production in recombinant *K. pneumoniae* strains. Kp(pTAC-*puuC*): Kp(pTAC) derivative expressing PuuC under *tac* promoter; Kp(pLAC-*puuC*): Kp(pLAC) derivative expressing PuuC under *lac* promoter. **(A)** Schematic diagram of vector construction. **(B–H)** Time courses of the biomass, glycerol consumption, and the production of 3-hydroxypropionic acid and byproducts in recombinant strains Kp(pTAC-*puuC*) and Kp(pLAC-*puuC*).

In this study, we started the construction of recombinant strains with two distinct promoters. The inducible *tac* promoter and constitutive *lac* promoter were tested for their activities to drive PuuC expression in *K. pneumoniae* (Fig. 2A), and accordingly the two recombinant strains named *K. pneumoniae*(pTAC-*puuC*) and *K. pneumoniae*(pLAC-*puuC*) were engineered. Colony PCR, restriction digestion of vectors, and DNA sequencing showed that the vectors pTAC-*puuC* (harnessing *tac* promoter) and pLAC-*puuC* (harboring *lac* promoter) were correctly constructed and transformed into *K. pneumoniae*. The gene *puuC* on vectors pTAC-*puuC* and pLAC-*puuC* showed 100% homology with the reported sequence of the *puuC* in the GenBank, suggesting that *puuC* gene was correctly cloned. By using flask shake cultivation, the two recombinant strains showed similar level of cell growth (Fig. 2B) and acetic acid production (Fig. 2E), although they consumed different amount of glycerol (Fig. 2C). Surprisingly, compared with the strain *K. pneumoniae*(pLAC-*puuC*), *K. pneumoniae*(pTAC-*puuC*) produced more 3-HP but less lactic acid, 1,3-PD, and 2,3-butanediol (2,3-BD) in 24 h fermentation (Fig. 2D–H). Overall, *tac* promoter was more suitable for the production of 3-HP than *lac* promoter.

**Figure 3:**
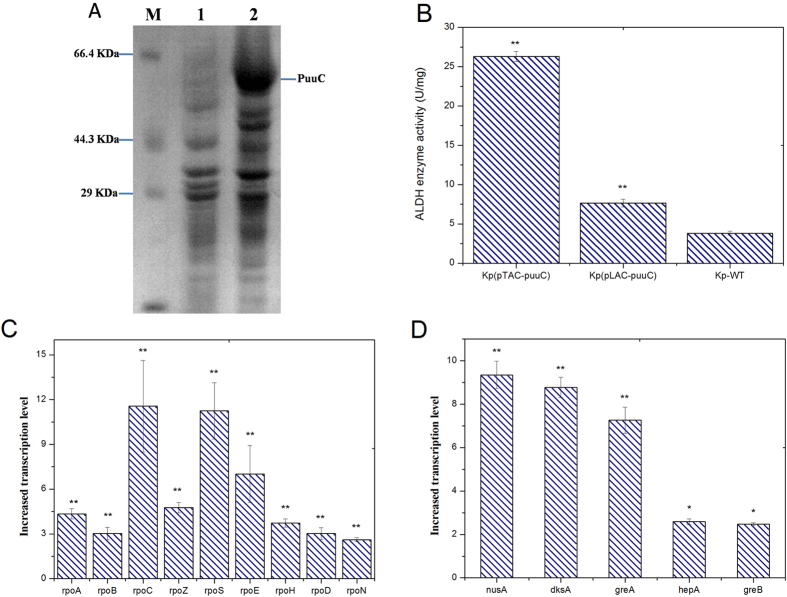
Performance of *tac* promoter in recombinant *K. pneumoniae* strains. **(A)** SDS-PAGE analysis of PuuC expression. M: protein marker; lane1: recombinant *K. pneumoniae* harboring empty vector pTAC; lane 2: PuuC expression in strain *K. pneumoniae*(pTAC-*puuC*). **(B)** ALDH enzymatic activities under distinct promoters. Kp-WT, wild type *K. pneumoniae*. **(C**) Relative transcription levels of RNA polymerase in *K. pneumoniae*(pTAC-*puuC*). GenBank accession numbers: *rpoA*: KPN_03695; *rpoB*: KPN_04365; *rpoC*: KPN_04366; *rpoZ*: KPN_03997; *rpoS*: PN_03103; *rpoE*: KPN_02898; *rpoH*: KPN_03827; *rpoD*: KPN_03474; *rpoN*: KPN_03612; **(D)** Relative transcription levels of RNA polymerase-related genes. GenBank accession numbers: *nusA*: KPN_03577; *dksA*: KPN_00145; *greA*: KPN_03591; *hepA*: KPN_00058; *greB*: KPN_03776. *P < 0.05, **P < 0.01 vs. control. In Fig. (**B**), Kp-WT was the control; In Fig. (**C,D**) the control was considered as 1. Error bar represents standard deviation of three independent experiments.

Next, we investigated PuuC expression through SDS-PAGE analysis. As shown in Fig. 3A, a significant band of approximately 56 KDa similar to the size of PuuC was appeared in lane 2, indicating that PuuC was successfully expressed and *tac* promoter functioned efficiently in *K. pneumoniae*. In contrast, ALDH expression was not observed in *K. pneumoniae*(pLAC-*puuC*) and wild type *K. pneumoniae*, indicating the lower activity of *lac* promoter than *tac* promoter. To further investigate the promoter strength, PuuC activity was assayed. As shown in Fig. 3B, the overall ALDHs (including PuuC and other native ALDHs) in recombinant strain *K. pneumoniae*(pTAC-*puuC*) exhibited activity of 26.31 U/mg, whereas it showed only 7.66 and 3.82 U/mg in *K. pneumoniae*(pLAC-*puuC*) and wild type *K. pneumoniae*, respectively. The wild type *K. pneumoniae* showed ALDH activity due to the existence of native ALDHs. Overall, these results indicated that *tac* promoter could efficiently drive PuuC expression which in turn elevated PuuC activity.

To probe the influences of *tac* promoter on the transcription machinery in *K. pneumoniae*, quantitative real-time PCR was performed. As shown in Fig. 3C,D, the relative transcription levels of RNAP and other five closely related genes in *K. pneumoniae*(pTAC-*puuC*) increased by 2 to 11-fold compared with those in *K. pneumoniae*(pLAC-*puuC*). Of these, *rpoC* and *rpoS* were the most significant up-regulated, presumably ascribing to the preferred recruitment of *rpoC* and *rpoS* by *tac* promoter in transcription initiation. These results suggested that *tac* promoter worked efficiently in *K. pneumoniae*.

### Optimization of fermentation conditions

Fermentation medium was optimized to maximize the 3-HP production. The ameliorated medium is as follows: 5.1 g/L K_2_HPO_4_•3H_2_O, 1.95 g/L KH_2_PO_4_, 8 g/L (NH_4_)_2_SO_4_, 0.125 g/L MgSO_4_•7 H_2_O and 4 g/L yeast extract. Under the shake flask cultivation, the strain *K. pneumoniae*(pTAC-*puuC*) produced 0.65 g/L 3-HP with ameliorated medium, while it generated only 0.36 g/L 3-HP with the original medium^14^. In addition, dry cell weight (DCW) increased from 0.87 to 1.12 g/L, and lactic acid reached 2.72 g/L which was 1.06-fold increase compared with the control. Interestingly, although biomass increased, acetic acid still kept at a low level.

To determine the optimal IPTG concentration for 3-HP production, the strain *K. pneumoniae*(pTAC-*puuC*) was grown in medium containing IPTG at different concentrations ranging from 0.01 to 2 mM, and we found that there was no affect on 3-HP production although IPTG is toxic to cells. For instance, when the concentration of IPTG was 0.02 mM and 0.05 mM in the media, the 3-HP titers of the strain *K. pneumoniae*(pTAC-*puuC*) were 2.22 g/L and 1.98 g/L, respectively (Fig. S1). Moreover, the total concentration of 3-HP and lactic acid was maintained around 2 g/L during the entire fermentation process (Fig. S1), indicating that there was a competition between 3-HP and lactic acid for glycerol carbon flux. In view of the above findings, we conclude that the optimal concentration of IPTG for 3-HP production was 0.02 mM in fermentation medium.

As shown in Table 1, the production of 3-HP and lactic acid was largely affected by the pH value. When pH value was 8.0, *K. pneumoniae*(pTAC-*puuC*) produced 428 mM lactic acid and 447 mM 3-HP for 36 h fermentation (Table 1). With the decrease of pH values, the level of lactic acid declined markedly (from 428 to 7 mM), showing that lactic acid production was partially controlled by the pH value. In contrast, the acetic acid formation was less affected. As shown in Table 1, when pH values were 6.0, 7.0, and 8.0, the concentrations of acetic acid were 76 mM, 66 mM and 103 mM, respectively. Apart from lactic acid and acetic acid, other byproducts such as 1,3-PD and 2,3-BD were also analyzed. Their accumulation was affected by pH values. For instance, 1,3-PD reached 119 mM at pH 7.0, while it was only 57 mM at pH 6.0, indicating the obvious influence of pH value on 1,3-PD formation. Noticeably, 2,3-BD was only produced at pH value around 7.0. pH 7.0 was also considered as the appropriate pH value for 3-HP production because of high glycerol conversion to 3-HP. 3-HP titer and glycerol conversion ratio culminated in 36 h at pH 7.0. Collectively, pH value was a critical factor for 3-HP production.

**Figure 4:**
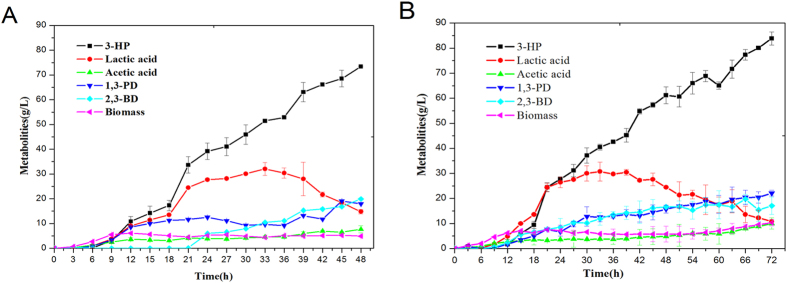
Fed-batch cultivation of recombinant *K. pneumoniae* strains in a 5 L bioreactor for production of 3-hydroxypropionic acid. **(A)** Time courses of metabolites production in *K. pneumoniae*(pTAC-*puuC*); **(B)** Time courses of metabolites production in *K. pneumoniae*Δ*ldh1*Δ*ldh2*Δ*pta*(pTAC-*puuC*). 3-HP: 3-hydroxypropionic acid; 1,3-PD: 1,3-propanediol; 2,3-BD: 2,3-butanediol. Biomass: dry cell weight.

To overproduce 3-HP, the fermentation in a 5 L bioreactor was extended to 48 h and pH value was maintained at 7.0 by supplying NaOH. The strain *K. pneumoniae*(pTAC-*puuC*) produced 73.4 g/L 3-HP in 48 h (Table 2), with 52.2% of glycerol conversion ratio and 1.53 g/L/h of 3-HP productivity. Surprisingly, nearly 30 g/L lactic acid was produced in 30 h, however it dropped to 15.69 g/L in 48 h (Fig. 4A). The fluctuation of lactic acid level may be attributed to two reasons: First, the active cell division at exponential growth phase and *tac*-driving PuuC overexpression necessitated lactic acid formation to provide both ATP and NAD^+^, which benefits cell growth and serves as the cofactor of ALDH, respectively; Second, cellular self-adjustment of pH value during late phase of fermentation and the artificial addition of NaOH remarkably decreased lactic acid level. As for the production of acetic acid, only 6.26 g/L was produced in 48 h. Furthermore, this strain also produced 17.9 g/L 1,3-PD and 20 g/L 2,3-BD (Table 2). Since 3-HP, 1,3-PD and 2,3-BD are valuable chemicals and their total production yields accounted for 81.7% of glycerol carbon flux, co-production of these compounds seems economically viable due to the maximized utilization of glycerol^20^,^21^.

### Optimization of metabolic flux

**Figure 5:**
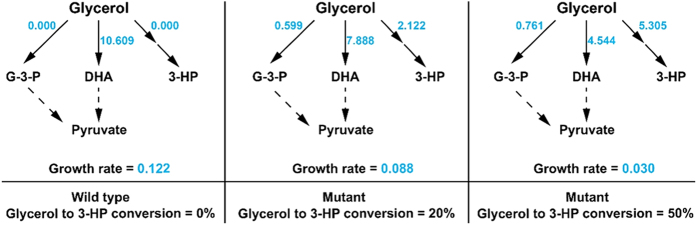
Proposed flux distribution model of glycerol metabolism in *K. pneumoniae.* Different conversion ratios of glycerol to 3-HP were used to investigate the flux distribution of glycerol in wild-type and mutant *K. pneumoniae*. Numbers in blue represent the *in silico* fluxes. DHA, dihydroxyacetone; 3-HP, 3-hydroxypropionic acid; G-3-P, glycerol-3-phosphate. The middle column indicates the mutant strain *K. pneumoniae*Δ*ldh1*Δ*ldh2* (pTAC-*puuC*), while the right column indicates the mutant strain *K. pneumoniae*Δ*ldh1*Δ*ldh2*Δ*pta*(pTAC-*puuC*).

In glycerol-based biosynthesis of 3-HP, lactic acid and acetic acid are major byproducts^19^. Although their formation benefits glycerol utilization by providing the cofactors and ATP, they account for a substantial portion of glycerol carbon flux. To divert more carbon flux to 3-HP, we deleted their synthesis genes *ldh1*, *ldh2* and *pta* by RecA homologous recombination. As shown in Fig. 4B, the mutant strain *K. pneumoniae*Δ*ldh1*Δ*ldh2*Δ*pta*(pTAC-*puuC*) produced 83.8 g/L 3-HP in 72 h with slightly compromised growth compared with *K. pneumoniae*(pTAC-*puuC*). This finding was consistent with the modeling result (Fig. 5, Table 3). Another noticeable finding was that, although *ldh1*, *ldh2* and *pta* genes were eliminated from the *K. pneumoniae* genome, the mutant strain *K. pneumoniae*Δ*ldh1*Δ*ldh2*Δ*pta*(pTAC-*puuC*) still synthesized lactic acid and acetic acid, indicating the presence of other pathways, e. g. amino acids pathways, for generating lactic acid and acetic acid.

### Proposed flux distribution model of glycerol metabolism

According to the observed high glycerol conversion ratio (46–54%) in 3-HP production, the flux distribution model of glycerol metabolism in recombinant *K. pneumoniae* was proposed by the *in silico* analysis. Similar to the experimental results, the flux of glycerol oxidation pathway and the cell growth rate were significantly reduced with the increase of 3-HP production (Fig. 5). In addition, glycerol kinase was recruited as the complementary pathway for supporting central carbon metabolism in the mutant strains. Thus, both the modeling and experimental results indicated the topological metabolic rigidity as well as the flexibility of the *dha* regulon in glycerol metabolism.

## Discussion

Here we developed an efficient system for biological production of 3-HP in *K. pneumoniae*. The success of this system attributed to the concert of promoters, key enzymes and fermentation processes. It should be noted that the IPTG-inducible *tac* promoter was shown to enable high expression of PuuC and thus 3-HP overproduction in *K. pneumoniae*. In a 5 L bioreactor, the recombinant *K. pneumoniae* strain overexpressing PuuC under *tac* promoter produced 73.4 g/L 3-HP in 48 h with 52% glycerol conversion ratio and 1.53 g/L/h productivity on glycerol. To reduce the formation of byproducts, the lactic acid and acetic acid synthetic genes were eliminated. Although the resultant mutant strain compromised growth (Fig. 5), it produced 83.8 g/L 3-HP in 72 h. To our knowledge, this is the highest 3-HP titer reported thus far. Below are the reasons behind this high production.

The first reason lies in the biochemical attributes of *K. pneumoniae.* In *K. pneumoniae*, glycerol conversion to 3-HP involves only two sequential reactions. Presumably, when glycerol is the sole carbon source, the 3-HP biosynthesis is actually the core metabolism because glycerol can be easily converted to 3-HP when a high-activity ALDH is available. In contrast, the 3-HP biosynthesis from glucose undergoes at least four reactions^10^. It is conceivable that manipulating multiple enzymes usually leads to metabolic flux imbalance and thus increases burden on the host. In addition to the difference of pathway steps, *K. pneumoniae* manifests striking attributes such as remarkable capacity to metabolize glycerol, active cell proliferation, and native ability to synthesize vitamin B_12_. These advantages empower *K. pneumoniae* to be a promising host for the production of 3-HP. For instance, since vitamin B_12_ is the cofactor of glycerol dehydratase, *K. pneumoniae* is obviously superior to *E. coli* which needs the addition of vitamin B_12_ to the fermentation medium. More importantly, the aggressive cell growth, powerful capability to metabolize glycerol, and high-activity of ALDH in *K. pneumoniae*, jointly provide a powerful driving force that pushes glycerol towards 3-HP biosynthesis. Although *Lactobacillus reuteri* also naturally produces 3-HPA^22^, its growth is significantly slower than *K. pneumoniae*, thereby increasing the production cost. In this study, *K. pneumoniae* grew actively due to vigorous consumption of glycerol. A total of 1553 mM glycerol was consumed with 82% glycerol conversion to valuable chemicals including 3-HP, 1,3-PD and 2,3-BD (Table 2, Fig. 4).

The second reason behind high 3-HP production is the effective expression system developed in this study. Previously, the lack of appropriate promoter restricts ALDH expression and accordingly hinders the conversion of 3-HPA to 3-HP. The importance of this study is the finding that *tac* promoter is functional in *K. pneumoniae*. This is evidenced by PuuC overexpression and enhanced PuuC activity (26.31 U/mg) compared with the recombinant strain recruiting *lac* promoter, whereby the PuuC activity was only 7.66 U/mg. Due to multiple copies of native ALDHs in *K. pneumoniae* genome, wild type *K. pneumoniae* also exhibited limited ALDH activity (3.82 U/mg) (Fig. 3B). To clarify the interplay between *tac* promoter and transcription machinery, quantitative analysis of RNAP was performed. We found that the transcription levels of RNAP and the five related genes were increased (Fig. 3C,D). RNAP is known to be consisted of sigma subunit and core enzyme which includes α, β, β′ and ω subunits forming a complex and encoded by *rpoA*, *rpoB*, *rpoC* and *rpoZ*, respectively^23^. It is well known that the *rpoC*-encoded β′ subunit mediates RNAP assembly. In this present study, we found that the *rpoC* gene in *tac*-driving strain showed notable transcription (Fig. 3C), indicating its likely involvement in RNAP assembly. Besides, sigma factors *rpoS* and *rpoE* were also strongly transcribed, implying that they may bind to *tac* promoter and initiate PuuC expression. In fact, *tac* promoter is hybridized by *trp* and *lac*UV5 promoters, and the affinity of *tac* with *rpoS* has been reported^16^. In view of this, we concluded that sigma factors might contribute to *tac* activity. Apart from RNAP, the five RNAP-related genes were also significantly transcribed. This can be explained by the fact that *nusA*, *greA* and *greB* govern transcription elongation. Another interesting finding in this study was that *dksA* showed similar transcription level to *rpoS* (Fig. 3D), which is consistent with the previous report that *dksA* induces *rpoS* at translational level^24^.

The third reason behind the high production of 3-HP may be the fermentation conditions, including IPTG concentration, pH value and medium composition. To maximize PuuC expression, IPTG concentration was optimized (Fig. S1). Compared with the IPTG concentrations used for triggering gene expression in other bacteria, only 0.02 mM IPTG (4.7 mg/L) was used in this study, clearly indicating low production cost. Indeed, 0.5 mM, 1 mM or 2 mM IPTG was usually used for *tac-*driving gene expression in *E. coli*^16^ and up to 5 mM IPTG were used for *tac-*driving gene expression in *Zymomonas mobilis* and *Pseudomonas putida*^17^,^25^. Due to the 3-HP accumulation in fermentation broth, pH value decreased and cell growth nearly ceased. To alleviate the stress of 3-HP, pH value was maintained at 7.0 by automatic addition of NaOH, which benefited cell growth and facilitated 3-HP formation. To further promote cell growth, fermentation medium was optimized, and the optimum medium contains a little more nitrogen sources compared with those previously reported (Fig. S2)^26^,^27^. Owning to above measures, both biomass and 3-HP titer were enhanced. Given that the PuuC activity (26.31 U/mg) was still relatively low compared with those previously reported^7^, 3-HP titer can be enhanced in the future work by taking a suite of measures, including employment of a high-activity ALDH, alleviation of feedback inhibition, and further optimization of fermentation conditions.

Collectively, all above results point to the fact that the biosynthesis of 3-HP depends on multiple factors, including cell growth, substrate provision, enzymatic activity, cofactor availability, redox balance, and cell tolerance to substrate and metabolites. For example, 3-HP production was shown to be closely coupled with cell proliferation especially at exponential phase^19^, and glycerol dissimilation is mediated by the *dha* regulon which steers parallel glycerol oxidation and reduction pathways. Interestingly, there exists a tradeoff between these two pathways. Intensifying the reduction pathway will simultaneously alter the flux toward oxidation pathway. Of the enzymes that execute glycerol dissimilation, DhaT, GDH, and DhaD, are three vital enzymes^10^. Their activities ascribe to multiple factors, including promoter strength, redox cofactors, substrate provision, and metabolite inhibition. Due to the buildup of metabolites including 3-HP, lactic acid and acetic acid, together with the increasing ionic strength, the cell tolerance to organic acids has emerged as a determinant for 3-HP production. Provided that the yield of metabolites could be considered as a quantitative trait controlled by multiple factors, it is conceivable that systematic optimization strategy is efficient for improving 3-HP production. Given the high 3-HP titer, high activity of *tac* promoter, low production cost, as well as novel insights into glycerol metabolism, we believe that this study is a big stride towards industrial production of 3-HP and polyesters consisting of 3-HP monomers^28^. More broadly, the efficient expression system developed here could be extended to the overproduction of 1,3-PD and 2,3-BD, both are top-valued bulk chemicals native to *K. pneumoniae*.

## Conclusions

In this work, we engineered recombinant *K. pneumoniae* strains expressing native ALDH (PuuC) under constitutive *lac* promoter or inducible *tac* promoter. *Tac* promoter outperformed *lac* promoter for the production of 3-HP in *K. pneumoniae*, which was evidenced by high expression of PuuC, the increased transcription of RNAP and related genes, and particularly the high ALDH activity towards 3-HPA. When a fed-batch culture was carried out under microaerobic conditions at pH 7.0 in a 5 L bioreactor, this *tac*-driving recombinant *K. pneumoniae* strain produced 73.4 g/L 3-HP with a cumulative yield of 52% on glycerol carbon and 1.53 g/l/h productivity in 48 h. Blocking the lactic acid and acetic acid synthesis genes slightly repressed cell growth, but elevated 3-HP titer to 83.8 g/L in 72 h. Given the efficient expression system and the obvious advantages of *K. pneumoniae* as a host, including super ability to utilize glycerol, natural ability to generate cofactor B_12_, and low consumption of IPTG, we believe that this work has substantially advanced biological production of 3-HP.

## Materials and Methods

### Strains, vectors and chemicals

Strains of *E. coli* Top10 and *K. pneumoniae* DSM 2026 were purchased from DSMZ GmbH, Germany. The vector pET-28a (Novagen) was employed in this study with minor modification. The original T7 promoter was replaced by *tac* promoter, yielding a new vector designated pTAC. Since *K. pneumoniae* is resistant to ampicillin, the original ampicillin resistance cassette in vector pUC19 was replaced by kanamycin resistance cassette to generate a new vector named pLAC. Taq DNA polymerase, restriction enzymes and T4 DNA ligase were purchased from TaKaRa (Dalian, China). Primer synthesis and DNA sequencing were accomplished by Biomed Co., Ltd. Other chemicals for enzymatic activity assay, gel electrophoresis, and HPLC analysis were products of Sigma.

### Construction of recombinants

The *puuC* gene (KPN_01018) was PCR-amplified from the genomic DNA of *K. pneumoniae*. The primers were listed in Table S1. The following are PCR procedures: initial denaturation at 94 °C for 4 min; followed by 30 cycles of 94 °C for 1 min, 55 °C for 45 s, 72 °C for 2 min; 72 °C for 10 min. The *puuC* gene was cloned into pTAC and pLAC, resulting in two recombinant vectors pTAC-*puuC* and pLAC-*puuC*, respectively. Transforming vectors into *K. pneumoniae* led to recombinant strains *K. pneumoniae*(pTAC-*puuC*) and *K. pneumoniae*(pLAC-*puuC*). The recombinant strains were grown in Luria-Bertani (LB) medium (NaCl, 10 g/L; peptone, 10 g/L; yeast extract, 5 g/L, additional 15 g agar per liter for solid LB medium). 50 μg/mL kanamycin was added to the medium for *K. pneumoniae*(pTAC-*puuC*), while 60 μg/mL ampicillin was added to the medium for *K. pneumoniae*(pLAC-*puuC*). The deletion of lactic acid and acetic acid synthesis genes *ldh1* (GenBank, KPN_01632), *ldh2* (GenBank, KPN_03949), and *pta* (GenBank, KPN_02688) followed the protocol of RecA homologous recombination. The recombinants were confirmed by colony PCR and sequencing.

### SDS-PAGE analysis

The medium used in this study was the same as the previously reported. After 3 h cultivation, IPTG was added to medium to induce *tac* promoter. Unlike *K. pneumoniae* (pTAC-*puuC*), *K. pneumoniae*(pLAC-*puuC*) does not need any inducer for PuuC expression because *lac* is a constitutive promoter. Strains were grown in 50 mL shake-flask containing 20 mL medium, rotated at 150 rpm for 12 h. Cell samples were harvested by centrifugation at 12000 rpm for 10 min, and incubated at 100 °C for 10 min with addition of bromophenol blue. The samples for gel electrophoresis were quantified by measuring cell concentration to ensure that all samples were equally used for analysis. SDS-PAGE analysis was carried out by using 12% polyacrylamide.

### Enzymatic activity assay

To measure ALDH activity, 10 mL cells were centrifuged at 10,000 rpm for 10 min and washed by 5 mL PBS (pH 7.0). Phenylmethanesulfonyl fluoride (PMSF) (10 mg/mL) was added to inhibit protease activity. The cells were sonicated and the resultant solution was centrifuged at 17,000 rpm for 15 min. Protein concentration was determined using Bradford reagent (BioRad). 200 μL cell-free fermentation broth was added into 5 mL Bradford solution. After 5 min reaction, absorbance was measured by spectrophotometer at 595 nm wavelength. Another 200 μL cell-free solution was added into 2 mL centrifugation tube containing 50 mM PBS solution, 10 mM 3-HPA and 0.2 mM NAD^+^, and subsequently incubated at 37 °C for 5 min. The amount of NADH was determined by measuring the increase of absorbance at 340 nm wavelength in spectrophotometer. One unit of enzyme activity was defined as the amount of enzyme used to generate 1 μmol NADH per minute.

### Real-time PCR analysis of transcription machinery

To monitor the influences of *tac* promoter on RNAP transcription, quantitative real-time PCR was performed. The strain *K. pneumoniae*(pTAC-*puuC*) was cultivated in an IPTG-containing medium, while the control strain was cultivated in the same medium only devoid of IPTG. A total of 9 genes, including four genes encoding RNAP subunits and five genes closely related to transcription, were analyzed for their transcriptional levels. Briefly, total RNA was extracted by Trizol method and further treated with RNase-free DNase I. Next, reverse transcription reaction was carried out to obtain cDNAs with GoScript™ Reverse Transcription System (Promega). Real-time analysis was performed in a gradient cycler (eppendorf, Germany) with SYBR Green (Roche) addition. All samples were tested in triplicate. The 16S rRNA coding gene in *K. pneumoniae* was used as the reference gene to determine the relative expression level of the subunits in RNAP.

### Shake-flask cultivation

The recombinant strains were grown in LB medium containing the following components per liter: 5 g yeast extract, 10 g NaCl, 10 g peptone, and 50 mg kanamycin. 1% of overnight culture was inoculated to the medium containing the same concentration of antibiotics. To maintain microaerobic conditions, the flasks were plugged with an oxygen-permeable cotton stopper and incubated in an orbital incubator shaker at an agitation speed of 180 rpm, 37 °C. Samples were taken out every 3 h to measure biomass, residual glycerol and 3-HP concentration. To optimize 3-HP-producing medium, the strains were grown in medium containing gradient concentrations of all ingredients except CaCl_2_ which was used to adjust initial pH value. Based on previous study, 0%, 25%, 50%, 100%, 150% and 200% of ingredients were individually tested. Meanwhile, ten gradient IPTG concentrations ranging from 0.01 to 2 mM were added to the medium to determine the most appropriate concentration for inducing PuuC expression. All examined samples were collected at 12 h.

### Bioreactor cultivation

Fed-batch cultivation of the strain was performed in a 5 L bioreactor (Baoxing, China) containing 3 L fermentation medium. The experiments were conducted in glycerol fed-batch mode. The fermentation conditions were similar to those previously reported^29^ with minor modification. The strain was pre-cultivated in 100 mL fermentation medium overnight at 37 °C and then added into the bioreactor. The agitation speed was 400 rpm and the air was supplied at 1.5 vvm. The temperature was 37 °C and pH value was adjusted to 6.0, 7.0 or 8.0 by addition of 5 M NaOH. Residual glycerol was maintained at 25 g/L. Dissolved oxygen (DO) was monitored automatically. Samples were collected every 3 h to examine cell concentration, residual glycerol, and metabolites.

### Analytical methods

Cell density was measured by using microplate reader at 600 nm with 200 μL fermentation broth added in a cuvette. The metabolites 3-HP, lactic acid and acetic acid were determined by high performance liquid chromatography (HPLC) system (Shimazu, Kyoto, Japan) equipped with a C_18_ column and a SPD-20 A UV detector at 210 nm. The column temperature was 25 °C, and mobile phase was 0.05% phosphoric acid at a flow rate of 0.8 mL min^−1^. 1,3-PD and 2,3-BD were quantitatively analyzed by HPLC (Shimadzu, Japan) equipped with a column of Aminex HPX-87 H Ion Exclusion particles (300 × 7.8 mm, Bio-Rad, Hercules, CA, USA) using a differential refractive index detector. The column was maintained at 65 °C and mobile phase was 5 mM sulfuric acid (in Milli-Q water) at 0.6 mL min^−1^. Residual glycerol concentration was measured every 3 h by a titration method with NaIO_4_ (for control of glycerol). All samples were filtered through 0.22-μm membrane filter.

### Metabolic flux modeling

Modeling analysis of glycerol metabolism was performed by using Flux Balance Analysis (FBA). The reconstructed model of *K. pneumoniae* MGH78578 iYL1228^30^ was modified to conduct all computations based on COBRA toolbox v2.0.5 within MATLAB_R2012b^31^. Glycerol was set as the sole carbon source and cell growth was used as the objective function. The uptake rates of glycerol and oxygen were referred to the reported value of 10.609 and 13.618 mmol.gDW^−1^, respectively^21^. And 10% oxygen uptake rate was used to simulate the microaerobic conditions. The flux distribution model of glycerol metabolism in *K. pneumoniae* was investigated by different conversion ratios of glycerol to 3-HP, and it was assumed there was no 3-HP production in wild-type *K. pneumoniae* during the simulation.

## Additional Information

**How to cite this article**: Li, Y. *et al.* High Production of 3-Hydroxypropionic Acid in *Klebsiella pneumoniae* by Systematic Optimization of Glycerol Metabolism. *Sci. Rep.* **6**, 26932; doi: 10.1038/srep26932 (2016).

## Supplementary Material

Supplementary Information

## Figures and Tables

**Figure 1:**
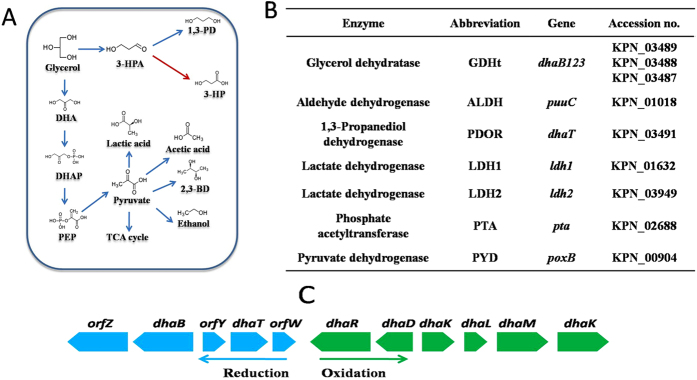
The *dha* regulon in *K. pneumoniae* . (**A**)Glycerol metabolic pathways; (**B**) Key enzymes participating in glycerol dissimilation; (**C**) Arrangement of enzyme genes on *K. pneumoniae* genome. 3-HPA, 3-hydroxypropionaldehyde. 1,3-PD, 1,3-propanediol. 3-HP, 3-hydroxypropionic acid. DHA, dihydroxyacetone. DHAP, dihydroxyacetone phosphate. PEP: phosphoenolpyruvate. 2,3-BD, 2,3-butanediol.

**Table 1:** Effects of pH value on carbon distribution in *K. pneumoniae*(pTAC-*puuC*) in bioreactor cultivation (36 h).

Product (nM)	3-HP	LA	AA	1,3-PD	2,3-BD	CG
pH 6.0	311	7	76	57	0	1360
GCR	23%	0.5%	5.6%	4.2%	0%	
pH 7.0	593	314	66	119	123	1300
GCR	46%	24%	5.1%	9.2%	9.5%	
pH 8.0	447	428	103	102	0	1090
GCR	41%	39%	9.4%	9.4%	0%	

GCR: glycerol conversion ratio; LA: lactic acid; AA: acetic acid; 1,3-PD: 1,3-propanediol; 2,3-BD: 2,3-butanediol; CG: consumed glycerol.

**Table 2:** Carbon distribution in *K. pneumoniae*(pTAC-*puuC*) in bioreactor cultivation (48 h).

Product	3-HP	LA	AA	1,3-PD	2,3-BD	Total
Titer (mM)	815	174	104	240	220	1553
Titer (g/L)	73.4	15.7	6.3	17.9	20	
GCR	52.2%	11.2%	6.7%	15.4%	14.1%	99.5%

GCR: glycerol conversion ratio. LA: lactic acid. AA: acetic acid. 1,3-PD: 1,3-propanediol. 2,3-BD: 2,3-butanediol.

**Table 3:** Carbon distribution in *K. pneumoniae*(pTAC-*puuC*)Δ*ldh1*Δ*ldh2*Δ*pta* in bioreactor cultivation (72 h).

Product	3-HP	LA	AA	1,3-PD	2,3-BD	Total
Titer (mM)	930	120	167	291	189	1716
Titer (g/L)	83.8	10.8	10.1	22.1	17.1	
GCR	54.1%	6.9%	9.9%	16.9%	10.8%	98.9%

GCR: glycerol conversion ratio. LA: lactic acid. AA: acetic acid. 1,3-PD: 1,3-propanediol. 2,3-BD: 2,3-butanediol.
