# Supplementary material for: High Production of 3-Hydroxypropionic Acid in Klebsiella pneumoniae by Systematic Optimization of Glycerol Metabolism
Source: Sci Rep. 2016 May 27;6:26932. doi: 10.1038/srep26932 (PMC4882505; doi:10.1038/srep26932)
Supplement: Supplementary Information [file srep26932-s1.doc]

**High Production of 3-Hydroxypropionic Acid in *Klebsiella pneumoniae* by Systematic Optimization of Glycerol Metabolism**

Ying Li^1,2^, Xi Wang^1^, Xizhen Ge^2^, Pingfang Tian^1^,*

^1^ Beijing Key Laboratory of Bioprocess, College of Life Science and Technology, Beijing University of Chemical Technology, Beijing 100029, People’s Republic of China;

^2^ College of Biochemical Engineering, Beijing Union University, Beijing 100023, People’s Republic of China

Ying Li, PhD

Beijing Key Laboratory of Bioprocess, College of Life Science and Technology, Beijing University of Chemical Technology, Beijing 100029, People’s Republic of China;

College of Biochemical Engineering, Beijing Union University, Beijing 100023, People’s Republic of China.

Xi Wang, PhD

Beijing Key Laboratory of Bioprocess, College of Life Science and Technology, Beijing University of Chemical Technology, Beijing 100029, People’s Republic of China.

Xizhen Ge, PhD, professor

College of Biochemical Engineering, Beijing Union University, Beijing 100023, People’s Republic of China.

Pingfang Tian, PhD, professor

Beijing Key Laboratory of Bioprocess, College of Life Science and Technology, Beijing University of Chemical Technology, Beijing 100029, People’s Republic of China.

**Supplementary figures**

**Fig. S1**. Effects of IPTG concentration on metabolites production.

Biomass: Dry cell weight; 3-HP: 3-hydroxypropionic acid.

**Fig. S2** Medium optimization.

Biomass: Dry cell weight; 3-HP: 3-hydroxypropionic acid.

**Table S1: Strains, vectors and primers used in this study.**

| Strain and plasmid | Description | Source |
| --- | --- | --- |
| **Strains** |  |  |
| *E. coli* Top 10 | Cloning host | Sunbiotech, China |
| *K. pneumoniae* DSM 2026 | Expression host and *puuC* gene donor | DSMZ, Germany |
| *K. pneumoniae*(pLAC-*puuC*) | *K. pneumoniae* DSM 2026 harboring *puuC* gene on vector pLAC | This study |
| *K. pneumoniae*(pTAC-*puuC*) | *K. pneumoniae* DSM 2026 harboring *puuC* gene on vector pTAC | This study |
| *K. pneumoniae*Δ*ldh1*Δ*ldh2*Δ*pta*(pTAC-*puuC*) | Mutant strain *K. pneumoniae* DSM 2026 harboring *puuC* gene on vector pTAC | This study |
|  |  |  |
| **Plasmids** |  |  |
| pET-28a(c+) | Expression vector; Kan^r^ | Novagen |
| pTAC | Expression vector; *tac* promoter; Kan^r^ | This study |
| pUC19 | Expression vector; Amp^r^ | NEB |
| pLAC | Expression vector; *lac* promoter; Kan^r^ | This study |
| **Primers** | Sequence (5’ to 3’) | Restriction enzymes |
| *puuC*, F | CGCGGATCCATGATGAATTTTCAGCACCTGGCTT | *Bam*H Ⅰ *tac* |
| *puuC*, R | CCCAAGCTTTCAAGACTCCAGGGCAATCCAGATG | *Hin*d Ⅲ |
| *puuC*, F | CCCAAGCTTATGATGAATTTTCAGCACCTGGCTT | *Hin*d Ⅲ *lac* |
| *puuC*, R | CGCGGATCCTCAAGACTCCAGGGCAATCCAGATG | *Bam*H Ⅰ |
| *Tac* promoter, F | GGAAGATCTTATATCCTTCTCCTTAAAGTTAAAC | *Bgl* Ⅱ |
| *Tac* promoter, R | CGCGGATCCCGATCCCGCGAAATTGACAATTAAT | *Bam*H Ⅰ |
| Kanamycin resistance gene, F | CGGGGTACCAACCCGGTAAGACACGACTTATCGCC | *Kpn* Ⅰ |
| Kanamycin resistance gene, R | TCCGAGCTCTTCCAAACTGGAACAACACTCAACCCTATC | *Sac* Ⅰ |

F: forward; R: reverse. The underlined sequence indicates restriction sites.
